# Enhancing Crop Yield and Adaptability through Sustainable Soil Management: Effective and Eco-Friendly Practices

**DOI:** 10.3390/plants13192798

**Published:** 2024-10-05

**Authors:** Dimitrios Bilalis, Ioannis Roussis, Ioanna Kakabouki

**Affiliations:** Laboratory of Agronomy, Department of Crop Science, Agricultural University of Athens, 11855 Athens, Greece; roussis@aua.gr (I.R.); i.kakabouki@aua.gr (I.K.)

The rapid growth of the global population has led to the construction of new residential colonies on fertile agricultural lands, resulting in a significant decline in crop production areas. Many biotic and abiotic stresses are also being attributed to abrupt changes in regional and global climates, which pose a threat to agricultural production. As a result, sustainable agricultural production is required on available cultivated lands. To maintain crop production, several effective and eco-friendly management practices are employed, including minimum/no tillage techniques, legume-based crop rotation, manuring, balanced fertilizer applications, cover crops, cropping patterns, irrigation techniques, etc. These techniques promote sustainable agriculture by ensuring sufficient production of high-quality crops.

This Special Issue (SI) aimed to gather articles focusing on recent scientific advances in soil fertility and crop production management to improve soil health, crop yield, and their adaptability under sustainable agriculture. The present SI is a collection of five articles (four research articles and one review article) covering various topics related to new agricultural practices that enhance our understanding of maintaining high soil fertility, new methods and technologies for crop fertilization, new sources and processes for providing high-quality crop products without adverse impacts on the soil, and the use of plant cover and/or crop associations.

The use of agroforestry systems, which combine woody perennial trees with herbaceous crops, is widespread in various regions worldwide, including Europe, South America, Africa’s Saharan region, and South Asia. Due to differences in climate and management practices, these systems come in diverse forms. Planting herbaceous crops between woody plants in alleys maximizes land and solar resource utilization in agroforestry systems [1]. Wang et al. [2] conducted an evaluative research study on the impact of intercropping on the soil–plant–atmosphere continuum using the SIMDualKc model, which achieves soil water balance with the dual Kc approach. Specifically, they focused on wolfberry (*Lycium barbarum* L.) sole-cropped and intercropped with alfalfa (*Medicago sativa* L.) and subjected to four different irrigation levels. They found that wolfberry intercropped with alfalfa and fully irrigated (75–85% θfc) decreased the annual mean soil evaporation by 32% compared to wolfberry monoculture under full irrigation. In particular, soil evaporation decreased from 292.48 mm in monoculture to 198.68 mm in the intercropping system. The intercropping system displayed a more effective canopy structure, reducing soil evaporation and partially relieving water stress compared to monocropping. Additionally, monocropping resulted in significantly higher soil moisture levels (1–15% higher) compared to intercropping regarding temporal dynamics, with the greatest difference occurring between the mid- and late stages of crop development, while differences were not statistically significant in the early stages.

Most soybean farmers in the Cerrado, the second biggest Brazilian biome, utilize direct seeding methods; thus, it is important to cultivate cash or cover crops during the off-season to protect the soil and promote the accumulation of organic matter, which directly affects the soil’s physicochemical characteristics [3]. In light of this, Alves et al. [3] conducted a field experiment in Jataí (Brazil) to examine the physicochemical features of the soil and the performance of soybean planted in different crop succession systems. They found that crop succession systems had no effect on yield in the two experimental years; however, the soybean–maize intercropped crop succession produced higher accumulated grain yields. These findings suggest that cover crops play a vital role in enhancing the physicochemical properties of soil over time. Despite this, the soybean–maize succession system is dominant in the Cerrado due to financial considerations at the expense of soil quality. The authors suggest that intercropping brachiaria (*Urochloa ruziziensis*) with maize could improve the soil physicochemical properties and increase yields in the long run.

In recent years, super-hybrid rice varieties with predominantly large panicles have had increasing crop yields. Nevertheless, as contrasted with multi-panicle-type varieties, large panicle-type varieties continue to confront yield stability issues, and there is little information available about their comparative advantages. To address this, Deng et al. [4] conducted a field experiment to assess the performance of large panicle-type hybrid rice (Y-liangyou 900, YLY900) and multi-panicle-type hybrid rice (C-liangyouhuazhan, CLYHZ) under different nitrogen treatments. They found that increased nitrogen fertilization had a greater impact on large panicle varieties. YLY900 presented a 6% higher average yield than CLYHZ owing to a greater number of spikelets per panicle. Additionally, the data from their study show that YLY900 demonstrated outstanding performance, as evidenced by higher light interception percentage (IP) and intercepted photosynthetically active radiation (IPAR) values, resulting in increased radiation use efficiency (RUE) and grain yield. They also found that delaying leaf senescence by increasing the leaf area index (LAI) at the post-heading stage for large panicle-type hybrid rice contributed to increased RUE, leading to higher biomass production and grain yield.

Floriculture crops are mainly grown in soilless environments and have a short production cycle. Soilless substrates typically do not contain enough mineral nutrients to sustain crop cycles, so additional applications of readily available fertilizers are often needed to produce marketable crops. This usually involves using water-soluble fertilizers through soaking or fertigation to provide essential elements rather than controlled-release fertilizers, which may not release enough nutrients throughout the short crop cycle. However, this often leads to nutrient leaching [5]. By implementing optimal management techniques in production decisions, such as adding fertilizer retention amendments to substrates or adjusting fertilization plans, nitrogen losses to the environment and associated costs can be reduced. Abdi et al. [6] investigated the effectiveness of using activated aluminum as a substrate amendment to maintain phosphorus (P) within containers and techniques to reduce phosphorus fertilization in *Tagetes* production over a six-week period. A commercial peat moss substrate preloaded with nutrients was treated with activated aluminum, allowing for comparisons between substrates with and without activated aluminum. When activated aluminum was added to commercial peat moss substrates that were treated with P throughout the entire six-week period, the leaching losses of P were substantially decreased by 89.5–97.7%. There was no difference in size or aboveground biomass among *Tagetes* plants regardless of the substrate or fertilizer management technique used. It was found that the addition of activated aluminum to substrates and/or the reduction in additional P inputs was effective in reducing the amount of P leached while maintaining the quality of *Tagetes*.

Tobacco (*Nicotiana tabacum* L.) has been cultivated for thousands of years to produce cigarettes, cigars, and other smoking products. However, due to its harmful effects on human health and the environment, the European Union has implemented strict laws to reduce tobacco smoking. Herbal cigarettes are often promoted as a healthier alternative to traditional tobacco cigarettes and are popular in Asian markets [7]. Despite some studies suggesting that herbal cigarettes are as harmful as tobacco cigarettes, introducing alternative crops to the European tobacco sector may facilitate the transition away from tobacco and smoking products. Mavroeidis et al. [8] conducted a comprehensive review of potential tobacco-alternative crops for use in the European smoking industry, outlining their advantages and disadvantages. The most common crops identified in the literature and in actual market products include Calendula (*Calendula officinalis* L.), mullein (*Verbascum thapsus* L.), ginseng (*Panax ginseng* C.A.Mey.), tea (*Camellia sinensis* (L.) Kuntze), chamomile (*Matricaria chamomilla* L.), and mentha (*Mentha* spp.). Although the aforementioned crops appear hopeful, the authors underlined that extended research is needed before they can be utilized in the European tobacco industry.

Together, these manuscripts provide valuable insights into the factors that affect crop productivity and quality. They also help us understand the impact of different cultivation practices and how the use of specific crop species, varieties, and soil management techniques can promote sustainable crop production.
